# Time-Restricted Eating as a Dietary Intervention for Polyendocrine Metabolic Ovarian Syndrome (PMOS): Protocol for a Randomized Controlled Trial

**DOI:** 10.3390/nu18213559

**Published:** 2026-10-28

**Authors:** Joanna Maria Pieczyńska-Zając, Sławomira Drzymała-Czyż, Ewa Pruszyńska-Oszmałek, Paweł Antoni Kołodziejski, Joanna Bajerska

**Affiliations:** 1 Department of Human Nutrition and Dietetics, Poznań University of Life Sciences, Wojska Polskiego 31, 60-624 Poznan, Poland; 2 Department of Bromatology, Poznań University of Medical Sciences, Rokietnicka 3, 60-806 Poznan, Poland; 3 Department of Animal Physiology and Biochemistry, Poznań University of Life Sciences, Wołyńska 35, 60-637 Poznan, Poland

**Keywords:** Time-Restricted Eating, Polyendocrine Metabolic Ovarian Syndrome, microbiota, neurotransmitters

## Abstract

Background: Time-restricted eating (TRE) appears to be a promising dietary strategy for women with polyendocrine metabolic ovarian syndrome (PMOS), with potential benefits for insulin resistance and metabolic health. Emerging evidence also suggests that TRE may be associated with changes in gut microbiota composition and microbial metabolites, as well as circulating biomarkers involved in metabolic regulation. However, the relationships among these factors remain poorly understood. Objective: The aim of this study is to test the hypothesis that adding TRE to Healthy Eating Plate recommendations is more effective in improving insulin resistance, assessed using HOMA-IR, than Healthy Eating Plate recommendations alone in women with PMOS. Exploratory analyses will investigate changes in gut microbiota composition, microbial metabolites, and circulating concentrations of neuroactive compounds. Methods: This two-arm, parallel-group randomized controlled trial will compare 8-h TRE with unrestricted meal timing in women aged 18 to 40 years with PMOS and a BMI of 25–40 kg/m^2^ with a 1:1 allocation ratio. Both groups will receive Healthy Eating Plate-based dietary advice and attend four visits; the TRE protocol will be followed for 8 weeks. The primary outcome will be a change in HOMA-IR from baseline to week 8. We will also assess changes in glucose and lipid metabolism, circulating neuroactive compounds, including GABA and kisspeptin, anthropometric parameters, hormonal profile, composition and diversity of the microbiota and its metabolites. Discussion: We anticipate that this study will expand the limited evidence regarding TRE in women with PMOS and provide exploratory insights into the relationships among gut microbiota composition, microbial metabolites, circulating GABA and kisspeptin, and insulin resistance. The findings may help identify potential mechanisms underlying the metabolic effects of TRE in this population. Protocol version: 1.1, August 2026. Trial registration number: NCT06204965. Trial status: Participant recruitment has not yet commenced.

## 1. Introduction

Polyendocrine Metabolic Ovarian Syndrome (PMOS), formerly known as Polycystic Ovary Syndrome (PCOS) [1], is a heterogeneous disorder characterized by reproductive, endocrine, and metabolic abnormalities. Under the Rotterdam criteria, PMOS encompasses several phenotypes that differ in the presence of hyperandrogenism, ovulatory dysfunction, polycystic ovarian morphology, insulin resistance, and cardiometabolic risk, a concept further emphasized by phenotype-based frameworks such as EGOI. Therefore, not all phenotypes necessarily share the same pathophysiological mechanisms or degree of metabolic impairment. Insulin resistance occurs particularly frequently in women with hyperandrogenic phenotypes, whereas normoandrogenic phenotypes may be characterized by a different metabolic profile [2]. Nevertheless, metabolic dysfunction, chronic low-grade inflammation, oxidative stress, and gut microbiota alterations are frequently reported in women with PMOS and may contribute to disease progression and long-term complications [3,4].

Although the pathophysiology of PMOS has not been fully elucidated, current evidence suggests that neuroendocrine dysfunction of the hypothalamic–pituitary–ovarian (HPO) axis plays a central role in its development. This dysfunction is characterized by altered gonadotropin-releasing hormone (GnRH) pulsatility, increased luteinizing hormone (LH) secretion, and excessive ovarian androgen production. Emerging evidence indicates that neuroendocrine mediators, including kisspeptin (KP) and γ-aminobutyric acid (GABA), may contribute to the regulation of GnRH neuronal activity and the neuroendocrine abnormalities observed in PMOS [5].

GABA, an important inhibitory neurotransmitter in the central nervous system [6], may participate in gut–brain communication through vagal and other neurohumoral pathways, thereby potentially influencing hypothalamic–pituitary–adrenal (HPA) axis activity and mood regulation [7]. In a study by Radwan et al., women with PCOS exhibited significantly lower serum GABA levels than healthy controls (*p* < 0.001). Serum GABA levels were also significantly negatively correlated with total testosterone (r = −0.30, *p* = 0.02), total cholesterol (TC) (r = −0.31, *p* = 0.01), and LDL cholesterol (LDL-C) (r = −0.23, *p* = 0.045) [8]. GABA has long been believed not to cross the blood–brain barrier; therefore, the potential effects of circulating GABA on brain function have been largely overlooked. However, emerging evidence suggests that changes in both circulating and brain GABA levels are associated with alterations in gut microbiota composition and may play an important role in modulating mental and metabolic health [9]. Several gut bacterial species are capable of producing GABA. Among them, Bifidobacterium, Lactobacillus, and Bacteroides are among the best-characterized GABA-producing genera, although circulating GABA levels cannot be considered a direct marker of microbial GABA production [9]. Furthermore, GABAergic signaling may influence peripheral tissues implicated in the pathophysiology of PCOS, including the immune system, the gut–brain axis, and the ovaries. Evidence from animal models and clinical studies in women with PCOS suggests that GABA supplementation may have beneficial effects on metabolic and reproductive health, including improvements in insulin sensitivity and modulation of sex hormone levels [10].

In turn, kisspeptin plays a critical role in activating the reproductive axis by stimulating pulsatile GnRH release through neural signaling to GnRH neurons, particularly during ovulation [11]. Moreover, a significant relationship was identified between increased kisspeptin concentrations and the occurrence of PCOS. These findings indicate that kisspeptin may have potential as a biomarker for the early detection of PCOS [5]. The results of Nandankar et al. suggest that a reduction in kisspeptin expression within KNDy neurons in females may contribute to the development of obesity by decreasing energy expenditure, without affecting food intake. Moreover, they indicated that KNDy-derived kisspeptin may play a sex-specific role in integrating reproductive and metabolic signals within the central nervous system [12]. These observations may be particularly relevant in PMOS, a condition characterized by the coexistence of reproductive and metabolic dysfunction.

Hypothalamic GnRH neurons serve as an important interface between metabolic and reproductive regulatory networks. Consequently, metabolic disturbances, including obesity, insulin resistance, and hyperinsulinemia, may alter GnRH neuronal activity and contribute to reproductive dysfunction in women with PMOS [13]. The gut–brain axis, a bidirectional communication network linking the gastrointestinal tract and the central nervous system, has also emerged as an important contributor to PMOS pathophysiology [14]. Gut microbiota-derived metabolites, including short-chain fatty acids (SCFAs), lipopolysaccharide (LPS), and GABA, are considered important mediators of this communication [15,16]. Consequently, gut dysbiosis associated with unhealthy dietary patterns and irregular meal timing may contribute to or exacerbate mechanisms involved in the development and progression of PMOS [17].

Given the emerging mechanistic links between gut microbiota and PMOS pathogenesis, dietary interventions targeting the gut microbiome represent a promising therapeutic strategy. Current dietary approaches for PMOS management primarily focus on energy restriction and low-glycemic-index dietary patterns, which have been associated with improvements in metabolic and reproductive outcomes. However, long-term adherence to these recommendations remains challenging for many women with PMOS [18]. The need for dietary strategies that are both effective and sustainable has therefore attracted increasing attention, particularly given the phenotypic heterogeneity of PCOS and the need to evaluate targeted lifestyle interventions aimed at improving metabolic abnormalities in women with PMOS.

Time-restricted eating (TRE) has been associated with favorable alterations in gut microbiota composition and diversity, including reported increases in the abundance of beneficial taxa such as Akkermansia and Faecalibacterium in both human and animal studies [19]. These microbiota alterations may contribute to some of the metabolic benefits associated with TRE and provide a potential mechanistic link between meal timing, gut microbiota, and PMOS-related outcomes.

Accumulating evidence suggests that TRE may be associated with improvements in selected metabolic and endocrine parameters relevant to PMOS. Clinical studies have reported favorable changes in endocrine and metabolic profiles, whereas preclinical studies further suggest potential benefits for ovarian morphology and folliculogenesis [20,21]. Consistent with these findings, a recent systematic review reported improvements in menstrual regularity and reductions in anti-Müllerian hormone levels among women with PMOS [20]. However, despite growing evidence for the metabolic benefits of TRE [22,23], it remains unclear whether TRE-induced alterations in gut microbiota composition, microbial metabolites, and circulating neurohormonal biomarkers are associated with improvements in PMOS-related outcomes.

To date, relatively few randomized controlled trials have investigated the effects of TRE on the gut microbiota and its metabolites [19]. To our knowledge, this will be among the first trials of TRE in women with PMOS to assess gut microbiota composition and microbial metabolites together with circulating GABA and kisspeptin and metabolic and reproductive hormone outcomes.

The trial follows a superiority framework, testing the hypothesis that adding TRE to Healthy Eating Plate recommendations will yield greater improvements in insulin resistance, assessed by HOMA-IR, than Healthy Eating Plate recommendations alone. Exploratory analyses will investigate associations of changes in gut microbiota composition, microbial metabolites, and circulating GABA and kisspeptin with changes in metabolic outcomes.

## 2. Materials and Methods

### 2.1. Study Setting

**Figure 1:**
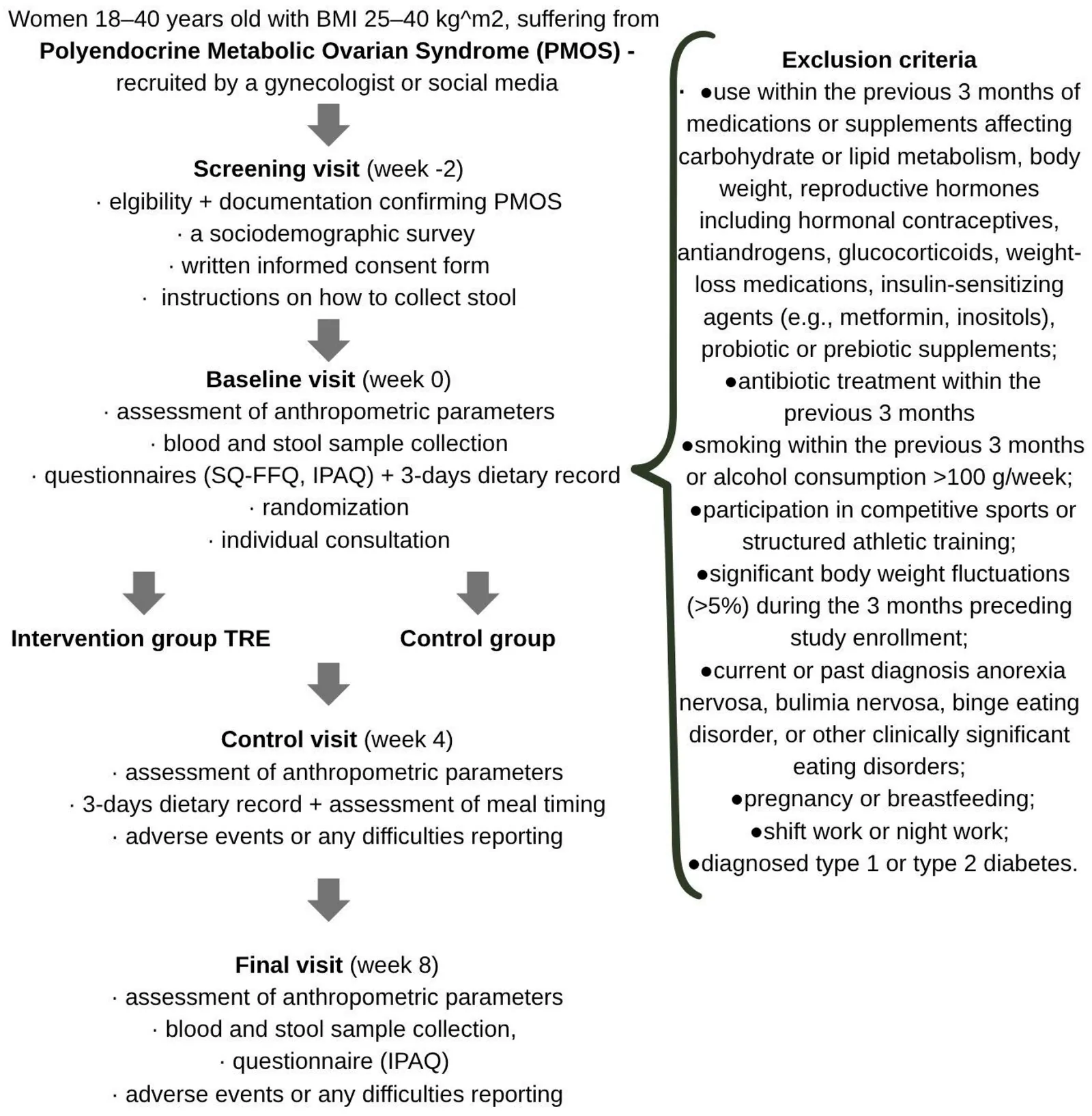
Flow chart presenting the individual stages of the study. PMOS—Polyendocrine Metabolic Ovarian Syndrome; SQ-FFQ—Semi-Quantitative Food Frequency Questionnaire; IPAQ—International Physical Activity Questionnaire.

This study is a two-arm, parallel-group randomized controlled superiority trial. Both groups will receive dietary advice based on the Healthy Eating Plate guidelines and will be allowed to eat ad libitum. The intervention group will additionally follow an 8-h TRE schedule, whereas the control group will not have any meal-timing restrictions. The experiment will be conducted at the Faculty of Food Science and Nutrition, Poznań University of Life Sciences. The study flowchart illustrating the individual stages of the study is presented in Figure 1. A detailed description of the procedures and individual stages is provided in the Study Design subsection.

### 2.2. Recruitment and Eligibility Criteria

#### 2.2.1. Operational Definition

In this protocol, PMOS is used as an umbrella term equivalent to PCOS diagnosed according to the Rotterdam criteria, consistent with the 2023 International Evidence-based Guideline. The term is used solely as an inclusive designation and does not imply any particular pathophysiological subtype described in emerging phenotype-based frameworks. Accordingly, no analyses or hypotheses related to subtype-specific effects are planned.

#### 2.2.2. Recruitment

In Poland, PMOS is typically diagnosed and managed in gynecological clinics. Participants will therefore be recruited through a collaborating gynecologist and through social media platforms including Facebook groups dedicated to women with PMOS, where study advertisements and contact forms will be posted. To prevent duplicate enrollment across recruitment pathways, all screened candidates will be recorded in a centralized screening log using unique participant identifiers.

#### 2.2.3. Ascertainment of Diagnostic Features

Irrespective of requirement pathway, the diagnosis will be verified by collaborating gynecologist on the basis of medical documentation, using the same predefined criteria for all participants. The research team will not perform independent diagnostic assessments. Each Rotterdam feature will be classified as present, absent, or not assessed, according to the following criteria: (i) ovulatory dysfunction: cycle length >35 days or <8 cycles/year; (ii) hyperandrogenism: clinical (modified Ferriman–Gallwey score ≥4) and/or biochemical (total testosterone and/or free androgen index above the laboratory reference range); (iii) PCOM: ≥20 follicles per ovary and/or ovarian volume ≥10 mL on transvaginal ultrasound or serum AMH above the assay-specific cut-off. Hormonal measurement obtained during hormonal contraceptive use, or within 3 months of its discontinuation, will not be accepted. Documentation must also confirm that alternative causes of hyperandrogenism and ovulatory dysfunction were excluded during the diagnostic work-up, including thyroid dysfunction, hyperprolactinemia, non-classic congenital adrenal hyperplasia, Cushing syndrome, androgen-secreting tumors.

#### 2.2.4. Incomplete Documentation and Phenotype Assignment

If documentation is insufficient, additional records will be requested from the treating physician. Participants with ≥2 documented Rotterdam criteria will be eligible, even if the third criterion is unavailable. Participants in whom PMOS cannot be confirmed will not be enrolled. Rotterdam phenotypes (A–D) will be assigned only when all three features are available; otherwise, phenotype will be recorded as indeterminate. Phenotype will not be used in subgroup or adjusted analysis.

#### 2.2.5. Eligibility

Eligible participants will be women aged 18–40 years with a BMI of 25–40 kg/m^2^ and a physician-established diagnosis of PMOS established according to the 2023 International Evidence-based Guideline for the Assessment and Management of Polycystic Ovary Syndrome [24]. The age range of 18–40 years was selected to minimize the inclusion of women undergoing the perimenopausal or menopausal transition, while the BMI range of 25–40 kg/m^2^ was defined to reduce metabolic heterogeneity within the study population and to increase the likelihood of detecting intervention-related changes in insulin resistance.

The exclusion criteria, assessed at the screening visit by structured interview and review of medical documentation, will be:

- Use within the previous 3 months of medications or supplements affecting carbohydrate or lipid metabolism, body weight, reproductive hormones including hormonal contraceptives, antiandrogens, glucocorticoids, weight-loss medications, insulin-sensitizing agents (e.g., metformin, inositols), probiotic or prebiotic supplements;
- Antibiotic treatment within the previous 3 months;
- Smoking within the previous 3 months or alcohol consumption >100 g/week;
- Participation in competitive sports or structured athletic training;
- Significant body weight fluctuations (>5%) during the 3 months preceding study enrollment;
- Current or past diagnosis of anorexia nervosa, bulimia nervosa, binge eating disorder, or other clinically significant eating disorders;
- Pregnancy or breastfeeding;
- Shift work or night work;
- Diagnosed type 1 or type 2 diabetes.

### 2.3. Randomization and Allocation

The randomization sequence will be generated by an independent researcher using a computer-generated randomization procedure with a 1:1 allocation ratio. Randomization will be stratified according to baseline BMI category (<30 kg/m^2^ vs. ≥30 kg/m^2^) to ensure a balanced distribution of overweight and obese participants across study groups. Within each stratum, permuted blocks of variable size (4 and 6, randomly selected) will be used, and a separate allocation sequence will be generated for each stratum. Block sizes will be known only to the independent researcher, responsible for generating the allocation sequence. Allocation concealment will be ensured using sequentially numbered, opaque, sealed, temper-evident envelopes, prepared independently of the recruitment team. Each envelope will contain the treatment allocation corresponding to a predefined participant identification number. Envelopes will be stored securely and opened strictly in numerical order within each stratum only after eligibility has been confirmed and written informed consent has been obtained. Following confirmation of eligibility and provision of written informed consent, the recruiting researcher will assign the participant the next study identification number within the appropriate BMI stratum and open the envelope bearing that number. The identification number will be used to link participant data throughout the study and will not disclose treatment allocation.

Due to the nature of the dietary intervention, blinding of participants and the researcher providing dietary consultations is not feasible. The recruiting researcher will have no role in outcome assessment.

The randomization list linking study identification numbers with treatment allocations will be securely retained by the independent researcher and will not be accessible to study personnel involved in recruitment, outcome assessment, or statistical analysis. Outcome assessors, including personnel performing DXA scans, collecting blood samples, and analyzing blood and stool samples, will remain blinded until the database is locked. Biological samples will be labeled with study identification numbers only. Statistical analyses will be performed on groups coded A and B, and the code will be released only after the primary analyses are complete.

### 2.4. Study Schedule

Participants will attend four study visits: a screening visit (week −2), a baseline visit (week 0), a follow-up visit after the first month of intervention (week 4), and a final visit at the end of the intervention period (week 8).

During the screening visit (week −2), participants will be assessed for eligibility by the principal investigator according to the predefined inclusion and exclusion criteria. Eligible participants will be asked to provide medical documentation confirming a physician-established diagnosis of PCOS, complete a sociodemographic survey, and sign a written informed consent form. They will also receive instructions and collection kits for stool sampling. Participants will be asked to complete a new 3-day food record (two weekdays and one weekend day) before each study visit throughout the intervention period.

Stool samples will be collected by the participants themselves within 24 h prior to the baseline visit, preferably from the first morning bowel movement, and delivered to the study site during the baseline visit with the completed food record. If delivery during the study visit is not possible, an alternative collection time will be arranged. During the baseline visit, each participant will undergo anthropometric assessment, including measurements of body weight, height, waist circumference and body composition. Fasting blood will also be collected. Participants will complete the food frequency questionnaire (SQ-FFQ) and the International Physical Activity Questionnaire (IPAQ). They will then be randomized (Section 2.3), informed of their allocation, and given an individual dietary consultation of approximately 15 min, though the duration may be adjusted to meet individual needs.

After four weeks of following the nutritional regimen, participants will attend a follow-up visit (week 4). Body weight and body composition will be assessed. Participants will submit a 3-day food record and a record of daily meal timing and will be asked about adverse events and difficulties in following the intervention. They will also receive a kit for use before the final visit.

Eight weeks after the intervention begins, participants will attend a final study visit. This visit will include anthropometric measurements, blood and stool collection, filling out the IPAQ again and delivery of a 3-day food record. Participants will also be asked again about any adverse events or difficulties experienced during the intervention.

### 2.5. Retention Strategies

Given the anticipated dropout in dietary interventions among women with polycystic ovary syndrome (31–63% over six months) [25], several retention strategies will be implemented in both groups. Participants will receive comprehensive oral and written instructions, sample recipes, and individualized dietary support to answer questions and help resolve difficulties. They will also receive reminder e-mails or text messages before scheduled study visits. The number of study visits will be limited to the minimum required by the protocol, and flexible appointment scheduling will be offered when needed. In the TRE group, the eating window may be adjusted by approximately ±1 h (e.g., due to work, study, or family commitments) to enhance feasibility while maintaining the intended intervention.

Adherence to TRE will be assessed using the recorded times of the first and last energy-containing eating occasions documented in 3-day food diaries completed at each follow-up visit. A day will be classified as compliant if all energy-containing eating occasions occurred within an 8-h window that started between 8:00 and 10:00 a.m. (adjustment of ±1 h as mentioned above). Days with no record will be classified as non-compliant. Adherence will be calculated as the percentage of compliant days during the 8 weeks intervention period. Participants achieving ≥80% compliant days will be included in the per-protocol analysis.

Adherence to the control condition will be assessed based on meal timing reported in three-day food diaries, with particular attention to whether participants unintentionally adopt a time-restricted eating pattern.

### 2.6. Adverse Events

An adverse event (AE) will be defined as any unfavorable or unintended sign, symptom, or health-related event occurring during the study period, regardless of whether it is considered related to the study intervention. AEs will be recorded in both groups and will include both solicited and spontaneously reported events. Solicited AEs will be assessed at the week-4 and week-8 visits using a standardized checklist covering hunger, headache, dizziness, weakness, excessive fatigue, nausea and other gastrointestinal symptoms, sleep disturbances, and menstrual cycle changes. Spontaneously reported AEs will comprise any other symptom or health concern raised by participants at visits or between visits by e-mail or telephone. A serious adverse event (SAE) will be defined as any AE that is fatal, life-threatening, requires hospitalization, results in persistent or significant disability, or is otherwise medically significant. All AEs and SAEs, regardless of their relationship to the intervention, will be documented and evaluated throughout the study. Each event will be assessed with regard to severity (mild, moderate, or severe), expectedness, and its potential relationship to the intervention (unrelated, possibly related, probably related, or definitely related). Participants will be instructed to report any symptoms or concerns via e-mail, telephone, or during scheduled study visits. Any SAE will be reported to the Bioethics Committee at Karol Marcinkowski Medical University in Poznań within 24 h of the investigators becoming aware. Although TRE is generally well tolerated [26] and no serious risks related to the 8-week intervention are anticipated, participants will be advised to seek appropriate medical evaluation and follow-up care if clinically relevant health concerns arise. The intervention will be discontinued in the event of pregnancy, an SAE considered related to the intervention, or signs of disordered eating. Participants may withdraw from the study at any time without consequences, and all withdrawals, discontinuations and reasons for discontinuation will be documented. After completion of the study, participants will receive the results of the examinations performed during the study, together with an explanation of the results during a final consultation. Throughout the study, participants may continue to receive care from specialist medical clinics where they are currently being treated. However, participants are required to inform the study team of any new medications or dietary supplements introduced during the study. Concurrent care provided by another dietitian and simultaneous participation in another medical experiment are not permitted during the study.

### 2.7. Interventions

Participants in both groups will receive dietary advice based on the Healthy Eating Plate model, to be followed ad libitum without prescribed energy restriction. They will be encouraged to fill half of their plate with vegetables and fruits (with an emphasis on vegetables), one-quarter with whole grains, and one-quarter with healthy protein sources, to prefer healthy plant oils, and to limit refined grains, processed foods, and sugar-sweetened beverages [27].

The control group will receive no instructions on meal timing, while participants in the TRE group will be instructed to consume all foods and energy-containing beverages between 9:00 a.m. and 5:00 p.m. (8-h eating window) and fast for the remaining 16 h until the following day (a 16:8 protocol), 7 days per week for 8 weeks. To support adherence, participants will be encouraged to install a free fasting reminder application on their mobile phones, such as Intermittent Fasting Easy Fast or Zero: Fasting & Food Tracker. A minor modification of the eating window up to one hour will be permitted to improve feasibility and adherence. During the fasting period, only non-caloric hot and cold beverages will be permitted (e.g., unsweetened coffee or tea, and water); all energy-containing products, including coffee cream, sweetened teas, alcohol, and snacks, will be prohibited.

In addition to oral instructions, both groups will receive printed educational materials on the principles of healthy eating and sample recipes, while only the TRE group will receive an additional leaflet on the principles of maintaining the eating window. The materials will be handed over during individual consultations after the group assignment has been revealed. Both groups will have the same number and timing of study visits. Because the TRE group will receive additional instructions, materials, and a reminder application, attention-related performance bias cannot be excluded.

### 2.8. Outcomes

Outcomes will be categorized as primary, secondary, and exploratory according to their clinical relevance, as shown in Table 1.

The schedule of outcome measurements is presented in Table 2 below.

#### 2.8.1. Anthropometry

Body weight will be measured in a standing position, without shoes and in light clothing, on an electronic BIA scale (Tanita MC-780 MA, Tanita, Tokyo, Japan) with an accuracy of 0.1 kg. Body composition, fat distribution, and bone density will be measured using dual-energy x-ray absorptiometry (DXA) with a Lunar ProdigyTM (GE Healthcare^©^, Madison, WI, USA, 2013), which uses the attenuation of ionizing radiation passing through tissues of varying densities. 

This method is non-invasive and widely used for body composition assessment. All DXA scans will be conducted by a single certified operator. Measurements will be obtained in the morning following an overnight fast. Daily quality control procedures will be carried out in accordance with the manufacturer’s instructions at each study visit. Participants will be required to remove all metal-containing clothing and accessories prior to scanning. The assessed body composition variables will include total body fat percentage (TBF) and total lean mass. Visceral adipose tissue (VAT) will be estimated using the enCORE™ software (version 17) with the CoreScan™ application (GE Healthcare, Madison, WI, USA). Reference values for body fat distribution and visceral adipose tissue will be based on the criteria reported by Ofenheimer et al. [28].

Waist and hip circumferences will be measured using a non-stretchable anthropometric tape. Waist circumference will be measured with the tape placed horizontally or slightly obliquely halfway between the lower edge of the ribs and the upper crest of the ilium. The measurement will be taken at the end of a normal expiration, with the participant standing upright and the abdomen relaxed. Hip circumference will be measured at the level of the greatest circumference of the buttocks, with the tape held horizontally and without compressing the soft tissue. Height will be measured to the nearest 0.1 cm using a stadiometer (RadWag, Radom, Poland).

#### 2.8.2. Biochemical Parameters

Participants will be classified according to Rotterdam phenotypes (A–D) based on the presence of hyperandrogenism, ovulatory dysfunction, and polycystic ovarian morphology (PCOM), assessed in accordance with current guidelines. Where appropriate, anti-Müllerian hormone (AMH) levels may be used as an alternative marker of PCOM. In this study, PMOS refers to the overall population of women meeting the Rotterdam diagnostic criteria, while the Rotterdam phenotypes are used to distinguish between clinically heterogeneous subgroups within this population. The prevalence of insulin resistance, defined as HOMA-IR ≥2.5, will be reported descriptively as part of the baseline characteristic.

Due to the high prevalence of oligomenorrhea and amenorrhea in women with PCOS, blood collection will not be restricted to a specific menstrual cycle phase. All samples will be collected in the morning after an overnight fast, and menstrual status at the time of sampling will be recorded and taken into account during data interpretation.

A total of 18 mL of venous blood will be collected into fluoride-oxalate tubes for glucose determination, serum separator tubes (SSTs) with clot activators for biochemical and hormonal analyses, and EDTA tubes for molecular analyses. Serum will be obtained by centrifugation after clot formation.

Total cholesterol (T-C), high-density lipoprotein cholesterol (HDL-C), triglycerides, and fasting glucose (FG) concentrations will be determined using standard enzymatic colorimetric methods on an automated biochemical analyzer in a certified external diagnostic laboratory. Low-density lipoprotein cholesterol (LDL-C) will be calculated using the Friedewald equation: LDL-C = total cholesterol − HDL-C − triglycerides/5 (mg/dL), when triglyceride concentrations are within the valid range for this calculation.

Insulin resistance will be evaluated using the Homeostasis Model Assessment Method. The HOMA-IR (Homeostatic Model Assessment of Insulin Resistance) [29] will be calculated using the following formula:

HOMA − IR = fasting glucose [mmol/L] × fasting insulin [μIU/mL]/22.5

The prevalence of insulin resistance in the study population will be reported descriptively as part of baseline characteristics.

McAuley index (MCAi) [30] will be calculated as follows:

MCAi = exp (2.63 − 0.28 × ln(fasting insulin [μIU/mL]) − 0.31 × ln(fasting triglycerides [mmol/L])

Castelli’s Risk Index I (CRI-I) [31] will be calculated as follows:

CRI-I = total cholesterol [mg/dL]/high-density lipoprotein [mg/dL]

Insulin (INS), follicle-stimulating hormone (FSH), luteinizing hormone (LH), testosterone, sex hormone-binding globulin (SHBG), kisspeptin, and gamma-aminobutyric acid (GABA) concentrations will be measured by enzyme-linked immunosorbent assay (ELISA). Serum GABA and kisspeptin concentration will be treated as exploratory peripheral biomarkers and should not be interpreted as direct measures of central GABAergic signaling or hypothalamic kisspeptin activity. All ELISA kits will be obtained from DiaMetra S.r.l., Spello, Italy (INS ELISA kit) or SunRed Biological Technology Co., Ltd., Shanghai, China (other ELISA kits).

These analyses will be performed using analyzers and equipment available at the Department of Animal Physiology and Biochemistry, Poznań University of Life Sciences. Biological material will be stored at −80 °C for future analysis related to the aims of the project, subject to participants consent and applicable regulations.

#### 2.8.3. Intestinal Microbiota and Metabolites

Participants will be asked to provide stool samples at baseline and at the final visit. Participants will receive a stool collection kit containing a transport container with semi-solid Amies or Stuart transport medium. Detailed instructions on sample collection, storage, and transport will be provided during the screening visit. Immediately after defecation, the stool sample should be transferred into the provided container containing the transport medium. Samples will not be frozen at any stage prior to analysis. Following collection, the sample should be kept refrigerated (2–8 °C), including during transportation to the study site. Participants will be advised to transport the sample in a cooler bag with an ice pack or in an additional container with ice. Stool samples should preferably be delivered to the study site and analyzed within 24 h of collection. Under exceptional circumstances, a maximum interval of 48 h between sample collection and analysis will be permitted, provided that the sample has been continuously maintained at 2–8 °C. Upon receipt, samples will be processed and analyzed as soon as possible. Any pre-analytical deviations, including failure to transfer the sample immediately into the transport medium, interruption of the cold chain, storage or transport temperatures exceeding 8 °C, or a time interval exceeding 48 h from collection to analysis, will be documented and evaluated for their potential impact on sample quality.

Bacterial DNA will be isolated from the provided stool samples using the QIAmp Fast DNA Stool Mini Kit. Then, the DNA will be properly secured and sent to an external company, Genomed S.A. (Warsaw, Poland), where microbiota assessment will be carried out using 16S rRNA gene sequencing targeting the V3-V4 region. Serum lipopolysaccharide (LPS) concentrations will be assessed using an enzyme-linked immunosorbent assay (ELISA). Quantitative and qualitative determination of short-chain fatty acids in feces will be performed by gas chromatography with flame ionization detection (GC-FID). The research will be carried out at the Department of Bromatology, Poznan University of Medical Sciences.

#### 2.8.4. Dietary Intake

Dietary intake will be assessed using a validated 72-item Semi-Quantitative Food Frequency Questionnaire (72-item SQ-FFQ) available on the DietTools4U platform [32] and a 3-day dietary record (two weekdays and one weekend day). The use of these complementary methods will enable both the assessment of habitual dietary patterns and the detailed evaluation of recent food intake.

The 72-item SQ-FFQ is based on a food-frequency approach and enables the assessment of habitual dietary intake, eating habits, and overall diet quality during the previous 12 months. Therefore, FFQ-derived dietary data will be used to characterize habitual pre-intervention dietary patterns and will be analyzed separately from dietary intake recorded during the intervention period. Participants will report the frequency of consumption of selected food and beverage groups over a specified reference period. Responses will be processed according to the standardized DietTools4U methodology to calculate dietary indicators and assess adherence to healthy eating recommendations. The questionnaire has been validated in the Polish population and demonstrated satisfactory reliability and validity, ensuring standardized and comparable dietary assessment while minimizing measurement bias [32]. The resulting data will be considered representative of habitual pre-intervention intake and will not be interpreted as a measure of dietary intake during the intervention period.

A three-day food diary will allow respondents to record all foods and beverages consumed over a specific period of two weekdays and one weekend day. A dietitian will provide written instructions via email to improve the accuracy of the food records. The instructions will explain how to accurately record real-time food intake, including the name of the food, the amount consumed using standard household measurements, preparation methods, brand names of commercially available products, and recipes for composite dishes. Instructions for using paper-based or web-based photographs (available at http://www.ilewazy.pl/ (accessed on 1 September 2026)) of food products and dishes will also be provided, along with comprehensive written guidance on dietary data collection. After completion, the dietitian will review the food records with each respondent to identify and clarify any omissions or ambiguities and make corrections where necessary. Reported amounts of foods and beverages will then be converted to grams and milliliters, respectively, and entered into the Dieta 6.0 dietary analysis software (National Food and Nutrition Institute, Warsaw, Poland) to determine the daily energy intake (kcal/d) and nutrient intake of the participants’ daily dietary intake.

#### 2.8.5. Physical Activity Level

Physical activity level (PAL) will be assessed using the short version of the International Physical Activity Questionnaire (IPAQ) by Biernat et al. [33] which has been officially translated and registered for use in Poland and enables classification of physical activity into internationally standardized MET-minute categories. On this basis, respondents are classified into one of three physical activity categories: low (below 600 METminutes/week), sufficient (600–1500 or 600–3000 METminutes/week) and high (above 1500 or 3000 METminutes/week).

### 2.9. Statistics

#### 2.9.1. Sample Size

The study was powered based on HOMA-IR. The sample size was calculated using G*Power software (RRID: SCR_013726, Version 3.1.9.6) based on data presented in Feyzioglu et al. 2023 [34], who reported a decrease in HOMA-IR from 4.29 ± 1.82 to 2.87 ± 0.98 in women with PCOS following TRE, corresponding to a standardized effect size of d = 0.97. For a two-sided, two-sample t-test with α = 0.05, 29 participants per group provide 95% power to detect an effect of this size. Allowing for 30% attrition, 42 participants per group (84 in total) will be enrolled.

Reliable effect-size estimates for gut microbiota and microbial metabolite outcomes were not available from previous randomized trials in women with PMOS. Therefore, HOMA-IR was selected as a clinically relevant metabolic outcome consistently reported in previous TRE studies. The effect size was based on an estimated within-group pre–post change and may overestimate the true between-group difference. It was used because no reliable between-group estimate for HOMA-IR was available in women with PMOS and should not be interpreted as an established minimal clinically important difference. Gut microbiota and microbial metabolite outcomes are exploratory and the trial is not powered to detect differences in these outcomes.

#### 2.9.2. Statistical Analyses

The primary analysis will follow the intention-to-treat (ITT) principle. All randomized participants will be analyzed in their allocated group regardless of adherence or study completion. A secondary per-protocol (PP) analysis will include participants who complete the week-8 assessment and meet the pre-specified adherence criterion (≥80% of days compliant in the TRE and control groups). The PP population will be defined by researchers not involved in the analysis before unblinding. The trial statistician will work with coded group labels (A and B).

Baseline metabolic characteristics will be summarized for the overall sample and separately by Rotterdam phenotype (A–D and indeterminate). The prevalence of insulin resistance (HOMA-IR ≥ 2.5) will be reported for the overall sample and separately for each Rotterdam phenotype as n (%). Anthropometric parameters and body composition, fasting blood glucose, fasting insulin, HOMA-IR, and relevant hormonal and lipid parameters will be presented as mean (SD) or median (IQR), as appropriate. Owing to the expected small numbers within phenotypes, these data will be reported descriptively only; no phenotype-based comparisons or subgroup analyses will be performed.

Baseline comparability between TRE and control groups will be assessed descriptively. Baseline characteristics of completers and non-completers will also be compared descriptively.

The primary estimand is the between-group difference in change in HOMA-IR from baseline to week 8 in all randomized participants, regardless of adherence to or discontinuation of the assigned intervention. Secondary estimands are defined analogously. Outcomes will be analyzed using linear mixed-effects models including all available measurements: baseline and week 8 for blood derived outcomes and additionally the interim visits for outcomes assessed at those visits. Group, time (categorical), and the group × time interaction will be included as fixed effects, baseline BMI as a covariate, and participant as a random intercept. Baseline measurements will enter the model as outcomes, so that all randomized participants contribute.

The treatment effect is the group × time contrast at week 8, reported as the estimated between-group difference in change from baseline with its 95% CI. HOMA-IR and other right-skewed outcomes will be log-transformed, and effects will be reported as ratios of geometric means. Model assumptions will be assessed using residual diagnostics.

The mixed-effects models include all randomized participants with available outcome data and provide valid estimates under the missing-at-random (MAR) assumption. Robustness of the primary result to departures from MAR will be assessed by delta-adjusted multiple imputation (tipping-point analysis), in which imputed week-8 values in participants with missing data are shifted progressively until the study conclusion changes. A complete-case analysis will be reported as a supplementary analysis.

Two-sided *p*-values < 0.05 will be considered statistically significant. No adjustment is needed for the single primary outcome. Secondary outcomes will be reported with 95% CIs without adjustment and interpreted as supportive. For exploratory microbiome analyses, the false discovery rate will be controlled with the Benjamini–Hochberg procedure applied separately within two predefined families: (i) taxon abundance and diversity measures, and (ii) microbiota-derived metabolites and related biomarkers.

The stool samples collected at baseline and week 8 will be analyzed. ASVs not assigned to a phylum will be removed, as will taxa that do not reach a relative abundance of 0.25% in at least one sample, to limit the influence of rare features susceptible to technical noise. α-Diversity (Shannon and Simpson indices, which weight evenness and dominance differently) will be calculated from ASV counts and analyzed with the mixed-effects model described above. β-Diversity will be assessed using Bray–Curtis dissimilarities, visualized by principal coordinates analysis, and tested for the group × time interaction using PERMANOVA (9999 permutations restricted within participants). As exploratory analyses, differential abundance of taxa at the phylum and genus levels will be evaluated based on within-participant changes from baseline to week 8 using the Mann–Whitney U test, with Benjamini–Hochberg adjustment for multiple comparisons within each taxonomic level. Analysis will be performed in R (version 4.0.3 (2020-10-10)) using the packages lme4 (version 1.1.29), lmerTest (version 3.1.3), emmeans (version 1.8.2), mice (version 3.14.0), phyloseq (version 1.34.0), microbiome (1.12.0), and vegan (version 2.6.2).

## 3. Ethics and Dissemination

This study was approved by the Bioethics Committee at Karol Marcinkowski Medical University in Poznań in October 2023 [approval number 646/23]. Participants will also be informed that they can withdraw from the study at any time without any consequences, including health consequences. The team reserved the right to withdraw a participant if they reported starting medications listed in the exclusion criteria, became pregnant, or developed a chronic disease.

The study will be performed in accordance with Good Clinical Practice and the ethical principles set forth in the Declaration of Helsinki. The full research protocol can be found on https://clinicaltrials.gov/study/NCT06204965 (accessed on 1 September 2026). Important protocol amendments will be submitted to the Bioethics Committee, updated in ClinicalTrials.gov, and communicated to investigators before implementation. Patients and members of the public will not be involved in the design, conduct, reporting, or dissemination plans of this trial. The study protocol was developed in accordance with the SPIRIT 2025 guidelines, and the completed checklist is available in the Supplementary Materials.

The results of this study will be published in international, peer-reviewed journals. Numerical data, statistical analyses, and graphical data will be deposited in the Open Data Repository RepOD, the institutional repository of PULS, and made publicly available upon publication, with a maximum 36-month embargo period if required by the publication process. Study data will be entered into a password-protected electronic database by authorized study personnel. Data quality will be monitored through range and consistency checks. All records will be pseudonymized using participant identification numbers and stored on secure institutional servers with regular backup procedures. Access to individual participant data (IPD) will be granted upon a justified request, subject to identity verification and approval by the principal investigator. Due to the low risk and relatively small scale of the study, an independent Data Monitoring Committee is not required; safety will be monitored by the principal investigator and the bioethics committee.

## 4. Discussion

In this paper, we present the protocol for an RCT testing whether TRE combined with Healthy Eating Plate dietary advice improves insulin resistance, measured by HOMA-IR, more than Healthy Eating Plate advice alone in women with PMOS. Exploratory analyses will examine changes in gut microbiota composition, microbial metabolites, and circulating GABA and kisspeptin.

PCOS is a heterogeneous condition associated with a broad spectrum of metabolic, reproductive, and psychological disturbances. Women meeting the Rotterdam criteria may differ substantially in the degree of hyperandrogenism, ovulatory dysfunction, insulin resistance, obesity, and cardiometabolic risk, reflecting potentially distinct underlying pathophysiological mechanisms. Recent phenotype-based frameworks, such as the EGOI proposal, further highlight the possibility that PCOS encompasses biologically distinct subgroups with different metabolic and reproductive characteristics. Although these frameworks have not yet been universally adopted in clinical practice, they emphasize the importance of considering phenotypic variation when interpreting research findings. Accordingly, alterations in the gut microbiota, neuroendocrine disturbances, and metabolic dysfunction may contribute to disease manifestation to different degrees among affected women. Despite growing evidence supporting the involvement of these mechanisms, effective and sustainable dietary interventions for PMOS remain limited. This may be especially important because conventional dietary strategies recommended for women with PMOS, such as a hypocaloric diet, a low-fat diet, and a low glycemic load diet, often show limited long-term adherence [18]. One possible explanation is that TRE focuses primarily on restricting the timing of food intake rather than on requiring continuous calorie counting or modifying food choices. Ezpeleta et al. emphasize that the sudden rise in TRE’s popularity is most likely due to its simplicity and the fact that it does not require individuals to count calories to lose weight [35]. In fact, Thomas et al. reported greater success eating within their 10-h windows compared with eating at or below patients’ calorie goals [36]. These findings suggest that TRE may represent a potentially acceptable and sustainable dietary strategy for women with PMOS.

The 9:00 a.m.–5:00 p.m. eating window was selected as a standardized and physiologically plausible early-TRE schedule for the purpose of this trial. The choice of an eight-hour eating window was based on recent data from a systematic review indicating that time-restricted eating regimens with windows of 6 to 10 h are generally safe, well-tolerated, and associated with a variety of potential health benefits [37]. Furthermore, Li et al. reported that the magnitude of the fasting-related effect was particularly evident when the fasting period reached approximately 16 h [38]. While the timing of the eating window was selected based on evidence suggesting that early TRE (first meal before 10:30 a.m.) seemed to have a greater beneficial effect than late TRE on insulin resistance [37], which may be especially important for women with PMOS. Moreover, late eating may result in positive energy balance and increased obesity risk [39]. Recognizing the practical constraints associated with an early eating schedule, a limited adjustment of approximately one hour was prespecified to facilitate adherence while maintaining the overall early-TRE character of the intervention. Indeed, O’Neal et al. identified several socio-environmental barriers, including work schedules, social obligations, and family commitments, which may influence the degree of adherence to the regime [40].

The present study is designed not only to evaluate the metabolic effects of TRE but also to investigate its potential influence on gut microbiota composition, gut microbiota-derived metabolites, including short-chain fatty acids (SCFAs), and neurohormonal pathways involved in the pathophysiology of PMOS. To our knowledge, this will be among the first RCTs in women with PMOS to simultaneously evaluate gut microbiota composition, gut microbiota-derived metabolites, neurohormonal pathways, and clinical outcomes in response to time-restricted eating. A major strength of this study is that both intervention groups will receive identical Healthy Eating Plate recommendations, thereby reducing potential confounding related to dietary composition and facilitating evaluation of a time-restricted eating intervention. Moreover, it is planned to blind the researchers who will conduct statistical tests and body composition and laboratory analyses. Another advantage of this study is the collaboration with a gynecologist, who will be responsible for participant recruitment and eligibility assessment. The clinician’s involvement facilitates the assessment of the practical significance of the results obtained and increases the likelihood of their implementation in everyday medical practice. Velissariou et al., in a systematic review of the literature, noted that intermittent fasting, particularly TRE, shows potential as a non-pharmacological intervention to improve reproductive health in women with PMOS by targeting, i.e., insulin resistance. However, none of the included studies investigated the effect of this dietary regimen on the activity of bacterial metabolites and neurotransmitters [20]. The involvement of researchers experienced in microbiome biostatistics will facilitate robust multivariate analyses and strengthen interpretation of the relationships among dietary factors, gut microbiota, microbial metabolites, and clinical outcomes.

Although the study has several methodological strengths, some limitations should be acknowledged. First, blinding both the intervention and the data collection process was not feasible because the intervention consisted of a lifestyle programme, making it impossible to conceal group allocation from either the participants or the main researcher. Second, dietary behavior will be assessed primarily through self-report questionnaires, which are susceptible to desirability bias. Nevertheless, the majority of the questionnaires used have been previously validated and widely applied in similar research, suggesting that any potential bias is likely to have affected both the intervention and control groups to a comparable extent. Stool collection will be performed by participants, which may introduce variability in pre-analytical conditions. However, by applying a well-planned, robust protocol (providing detailed information and an appropriate sample container), this limitation will be significantly reduced. It is worth noting that the composition of the gut microbiota is influenced by numerous factors related to the host and the environment, including lifestyle, the use of medications, stress level and individual characteristics. Despite the application of appropriate exclusion criteria, the risk of residual confounding cannot be entirely ruled out.

An important limitation concerns the interpretation of circulating LPS, which should be considered an indirect marker of systemic endotoxin exposure that may reflect, among other factors, translocation of bacterial products from the gastrointestinal tract, rather than a direct measure of intestinal permeability [41]. Moreover, its measurement is challenging due to the low circulating concentrations of LPS, its molecular heterogeneity, interactions with host-derived components, and assay-specific characteristics [42].

A high drop-out rate resulting from psychological burden or low participant motivation may reduce statistical power [43]. This risk was considered during sample-size estimation and will be addressed through predefined retention strategies.

Another limitation of the study is the relatively short duration of the intervention (8 weeks), which restricts the ability to determine whether the observed effects of TRE on metabolic parameters and gut microbiota persist in the long term. Previous studies have shown that an 8-week TRF intervention was sufficient to detect changes in selected metabolic markers [44] and a 4-week intervention influenced gut microbiota composition and diversity [45]. Nevertheless, this duration does not allow conclusions regarding the long-term sustainability of the observed effects. Therefore, longer intervention periods are required to assess the durability and long-term clinical significance of these effects in women with PMOS. Moreover, a one-hour variation in the timing of the eating window may introduce some variability in the metabolic effects of meal timing, as earlier or later 8-h windows may differ in relation to circadian rhythms, sleep, and the timing of the last meal. However, the limited modification preserves the overall duration and early-TRE character of the intervention. The actual timing of the eating window will be recorded throughout the study to assess adherence and meal-timing variability and to account for these factors in the statistical interpretation of the results.

Furthermore, inclusion of women representing different Rotterdam phenotypes reflects the clinical heterogeneity of PCOS. Meeting the Rotterdam diagnostic criteria does not necessarily imply the presence of insulin resistance, as metabolic profiles may vary substantially across phenotypes and insulin resistance is not a diagnostic criterion of PMOS. Consequently, some participants with non-hyperandrogenic phenotypes may have normal baseline HOMA-IR values. Because insulin resistance is not an inclusion criterion, this heterogeneity may attenuate the overall effect of the intervention. Furthermore, the study is not designed or powered to evaluate phenotype-specific responses. Therefore, any observed benefit of TRE should be interpreted as an average effect across a phenotypically heterogeneous population, rather than as evidence of a common underlying metabolic mechanism or a uniform treatment response across phenotypes. Phenotype-specific baseline characteristics will be reported descriptively only to support interpretation of the findings and to inform future research.

We anticipate that this will be one of the largest studies assessing the effectiveness of time-restricted eating in women with PMOS, providing insights into associations between gut microbiota composition, microbial metabolites, circulating neuroactive biomarkers, and metabolic outcomes. Our trial will therefore contribute significantly to the body of knowledge regarding the effects of TRE in women with PMOS.

## Figures and Tables

**Table 1:** Outcome categories.

Primary Outcome	Secondary Outcomes	Exploratory Outcomes
Change in HOMA-IR	Change in glucose metabolism (FG, INS)	Changes in gut microbiota diversity and composition, SCFA concentrations, LPS levels, and other microbiome-related biomarkers.
	Change in lipid metabolism	
	Change in hormonal profile	
	Circulating GABA and kisspeptin concentrations will be evaluated as exploratory neurohormonal biomarkers	
	Change in anthropometric parameters (body weight, body composition, waist and hip circumference)	
	Change in energy consumption	

Abbreviations: SCFAs—Short-Chain Fatty Acids; LPS—Lipopolysaccharide; FG—Fasting glucose; INS—insulin; HOMA-IR—Homeostatic Model Assessment of Insulin Resistance; GABA—Gamma-Aminobutyric Acid.

**Table 2:** Schedule of outcome measurements.

Parameter	Screening Visit	Baseline Visit	Week-4 Follow-Up Visit	Final Visit
Assessment of sociodemographic factors	X			
Assessment of dietary intake (three-day food diary) and PAL		X		X
Anthropometric measurements		X	X	X
Biochemical parameters (lipid and carbohydrate metabolism, LPS, hormones) and circulating neurohormones (GABA, KP)		X		X
Composition of the intestinal microbiota in feces and concentration of SCFAs		X		X
Adverse events		X	X	X
Adherence to the regime		X	X	X

Abbreviations: PAL—Physical activity level; SCFAs—Short-Chain Fatty Acids, LPS—lipopolysaccharide; KP—kisspeptin; GABA—gamma-aminobutyric acid.

## Data Availability

The data will be deposited in the Open Data Repository RepOD, the institutional repository of PULS, and made publicly available upon publication, with a maximum 36-month embargo period if required by the publication process. Access to pseudonymized individual participant data (IPD) will be granted upon a justified request, subject to identity verification and approval by the principal investigator.

## Supplementary Materials

The following supporting information can be downloaded at: https://www.mdpi.com/article/10.3390/nu18213559/s1, SPIRIT 2025 editable checklist. Reference [46] is cited in the supplementary materials.
